# Integrin αvβ6 mediates epithelial-mesenchymal transition in human bronchial epithelial cells induced by lipopolysaccharides of *Pseudomonas aeruginosa* via TGF-β1-Smad2/3 signaling pathway

**DOI:** 10.1007/s12223-019-00728-w

**Published:** 2019-06-26

**Authors:** Weiming Liu, Tieying Sun, Yong Wang

**Affiliations:** 1 Department of Respiratory and Critical Care Medicine, Beijing Hospital, National Center of Gerontology, No.1 Dahua Road, Dong Dan, Beijing, 100010 China; 2 Graduate School, Peking Union Medical College, Beijing, China; 3 Graduate School, Chinese Academy of Medical Sciences, Beijing, China; 4 College of Basic Medicine, Chengdu University of Traditional Chinese Medicine, Chengdu, China

## Abstract

Lower respiratory tract infection due to *Pseudomonas aeruginosa* has become increasingly challenging, resulting in a worse morbidity and mortality. Airway remodeling is a common phenomenon in this process, to which epithelial-mesenchymal transition (EMT) may contribute as an important promoter. Previous studies showed that epithelium-specific integrin αvβ6–mediated EMT was involved in pulmonary fibrosis via transforming growth factor-β1 (TGF-β1) signaling, but whether integrin αvβ6 plays a role in the *P. aeruginosa*–associated airway remodeling remains unknown. BEAS-2B cells were incubated with lipopolysaccharide (LPS) from *P. aeruginosa* in the presence or the absence of integrin αvβ6–blocking antibodies. Morphologic changes were observed by an inverted microscopy. The EMT markers were detected using Western blotting and immunofluorescence. The activation of TGF-β1-Smad2/3 signaling pathway was assessed. Furthermore, matrix metalloproteinase (MMP)-2 and -9 in the medium were measured using ELISA. *P. aeruginosa*’s LPS decreased the expression of the epithelial marker E-cadherin and promoted the mesenchymal markers, vimentin and α-smooth muscle actin in BEAS-2B cells. The expression of integrin αvβ6 was significantly increased during EMT process. Blocking integrin αvβ6 could attenuate *P. aeruginosa*’s LPS-induced EMT markers’ expression via TGF-β1-Smad2/3 signaling pathway. Furthermore, blocking integrin αvβ6 could prevent morphologic changes and oversecretion of MMP-2 and -9. Integrin αvβ6 mediates epithelial-mesenchymal transition in human bronchial epithelial cells induced by lipopolysaccharides of *P. aeruginosa* via TGF-β1-Smad2/3 signaling pathway and might be a promising therapeutic target for *P. aeruginosa*–associated airway remodeling.

## Introduction

*Pseudomonas aeruginosa* is one of the most frequent opportunistic pathogens, responsible for serious lower respiratory tract infection in patients with cystic fibrosis (Moore and Mastoridis 2017), chronic obstructive pulmonary disease (Almagro et al. 2012), bronchiectasis (Sanchez-Munoz et al. 2016), and accounting for 17.8% of all isolates in hospital-acquired pneumonia (Ding et al. 2016). Due to severe drug resistance and limited therapeutic options, *P. aeruginosa*–caused lower respiratory tract infection is becoming difficult to treat, which often results in high mortality in these patients (Zhuo et al. 2008). Emerging evidence have revealed that *P. aeruginosa* is associated with airway remodeling (Botha et al. 2008; Vos et al. 2008; Cigana et al. 2016), which is characterized by aberrant repair of the epithelium and accumulation of fibroblasts and could give rise to irreversible decline of pulmonary function and poor prognosis. However, little is known how to alleviate airway remodeling associated with *P. aeruginosa*.

Recently, epithelial-mesenchymal transition (EMT) has been identified as an important source of fibroblasts that could contribute to the remodeling of the airways. EMT is a reversible process by which epithelial cells change the phenotype and function into that of fibroblast-like, mesenchymal ones, characterized by loss of epithelial markers, acquisition of mesenchymal markers, and matrix metalloproteinase production (Kalluri and Neilson 2003; O’Connor and Gomez 2014). EMT plays an important role in the process of fibrosis (Kim et al. 2006; Flier et al. 2010; Marmai et al. 2011); thus, to reverse EMT process may ameliorate airway fibrotic remodeling. Human bronchial epithelial cells are the first line of defense against invading organisms, which could undergo EMT when exposed to many stimuli (Doerner and Zuraw 2009; Gulino et al. 2016; Polimeni et al. 2016). However, whether EMT in human bronchial epithelial cells contributes to *P. aeruginosa*–associated airway remodeling is rarely studied.

Transforming growth factor-β1 (TGF-β1) is a pleiotropic growth factor which has been considered a key inducer of fibrosis and has a central role in regulating EMT process by mediating Smad-dependent and Smad-independent signaling pathways (Ask et al. 2008). Previous studies revealed that *P. aeruginosa* could significantly increase the secretion of TGF-β1 in vivo and in vitro (Yang et al. 2011), which suggested that TGF-β1 may play a critical role in *P. aeruginosa*–associated airway remodeling. Although the potential therapeutic value of TGF-β1 in fibrotic remodeling, however, blocking of TGF-β1 globally may result in severe side effects as previously reported (Shull et al. 1992; Kulkarni et al. 1993; Dickson et al. 1995). Thus, to regulate TGF-β1 activation selectively by inhibiting activation of TGF-β1, such as epithelium-restricted integrin αvβ6, may represent an ideal strategy.

Integrin αvβ6, an epithelium-restricted transmembrane protein, is expressed at an extremely low level in normal epithelial cells and dramatically increased in response to injury or inflammation stimuli, which could activate endogenous TGF-β1 in a paracrine-like manner. Blocking integrin αvβ6 could inhibit the local activation of TGF-β1 in fibrosis process without systemic side effects (Munger et al. 1999; Horan et al. 2008; Puthawala et al. 2008; Katsumoto et al. 2011). In addition, activation of Smad2/3 signaling pathway is found in fibrogenic process regulated by integrin αvβ6 (Wang et al. 2015). Thus, we hypothesized that EMT may get involved in *P. aeruginosa*–associated airway fibrotic remodeling, which could be regulated by integrin αvβ6 via activation of TGF-β1-Smad2/3 signaling pathway.

## Materials and methods

### Cell culture and treatment

BEAS-2B cells derived from the normal human bronchial epithelium were purchased from ATCC and cultured in bronchial epithelial cell growth medium (BEGM, Lonza) at 37 °C in a humidified 5% CO_2_ atmosphere. Culture flasks should be precoated with a mixture of 0.01 mg/mL fibronectin, 0.03 mg/mL collagen, and 0.001 mg/mL bovine serum albumin dissolved in the medium according to the ATCC recommended protocol. To induce EMT, BEAS-2B cells were seeded at 80% confluence a day before the stimulation of *P. aeruginosa*’s LPS (Sigma). BEAS-2B cells were pretreated with Integrin αvβ6–blocking antibody (10D5, Abcam) or TGF-β1-Smad2/3 signaling inhibitor, SB431542 (Cell Signaling), for 2 h prior to incubation with 2 μg/mL *P. aeruginosa*’s LPS (Gong et al. 2014). Morphologic images were captured with an Olympus Inverted Microscope.

### Western blotting analyses

Cells were lysed in RIPA buffer (SolarBio, 50 mM Tris/HCl, pH 7.4, 150 mM NaCl, 1% (*v*/*v*) NP-40, 0.1% (*w*/*v*) SDS) containing 1% (*v*/*v*) phenylmethylsulfonyl fluoride (SolarBio), 0.3% (*v*/*v*) protease inhibitor (Sigma Aldrich), and 0.1% (*v*/*v*) phosphorylated proteinase inhibitor (Sigma) and then clarified by centrifugation. The supernatant was collected and then separated on an SDS-PAGE gel (10% (*v*/*v*) polyacrylamide), transferred onto a PVDF membrane. Nonspecific binding was blocked in Tris-buffered saline with Tween 20 (TBS-T) with 8% (*w*/*v*) milk for 2 h. After incubation with primary antibodies against β-actin (Abmart), E-cadherin (E-Cad, Abcam), vimentin (Vi, Abcam), α-smooth muscle actin (α-SMA, Sigma), Smad2/3 (Abcam), and p-Smad2/3 (Abcam) for overnight at 4 °C, the membranes were washed with TBS-T for several times and incubated in HRP-linked secondary antibodies (Abmart) for 2 h at room temperature (RT). After repeated washing with TBS-T, the immunoreactive proteins were visualized using enhanced chemiluminescence (Millipore) according to the manufacturer’s instructions and quantified using density analysis, normalized against β-actin and expressed as the fold change compared with the control.

### Immunofluorescence

Cells grown on chamber slides were fixed in 4% paraformaldehyde for 30 min at room temperature and permeabilized with 0.1% Triton X-100 at RT for 5 min. The slides were washed with phosphate-buffered saline (PBS) three times and then blocked in 3% bovine serum album (BSA) for 60 min at RT. The cells were incubated with primary antibodies against human E-Cad and Vi (1:100 dilution in PBS with 1% BSA) for 2 h at RT. After several washes with PBS, the slides were incubated with Alexa Fluor 488–conjugated anti-rabbit IgG (Zhongshan Biotechnology, 1:100 dilution in PBS with 1% BSA) for 60 min at RT. After several washes for 15 min with PBS, the slides were incubated with Hoechst 33258 (10 μg/mL) for 10 min at RT. Finally, the slides were washed again, mounting reagent was added, and images were captured using a fluorescence microscope.

### ELISA

To determine the secretion of active TGF-β1, metalloproteinase (MMP)-2, and MMP-9, ELISA procedure was performed according to the manufacturer’s instructions (Beijing Rui’erxinde Technology).

### Statistical analysis

Data were expressed as mean ± SEM. Significance of the results were analyzed by performing one-way ANOVA, except time-dependent changes of the expression of EMT markers and integrin αvβ6 in BEAS-2B cells which were analyzed with repeated measures ANOVA test. Tukey’s post hoc tests were performed for multiple comparisons. *p* < 0.05 was considered significant.

## Results

### *P. aeruginosa*’s LPS-induced EMT in BEAS-2B cells

**Fig. 1:**
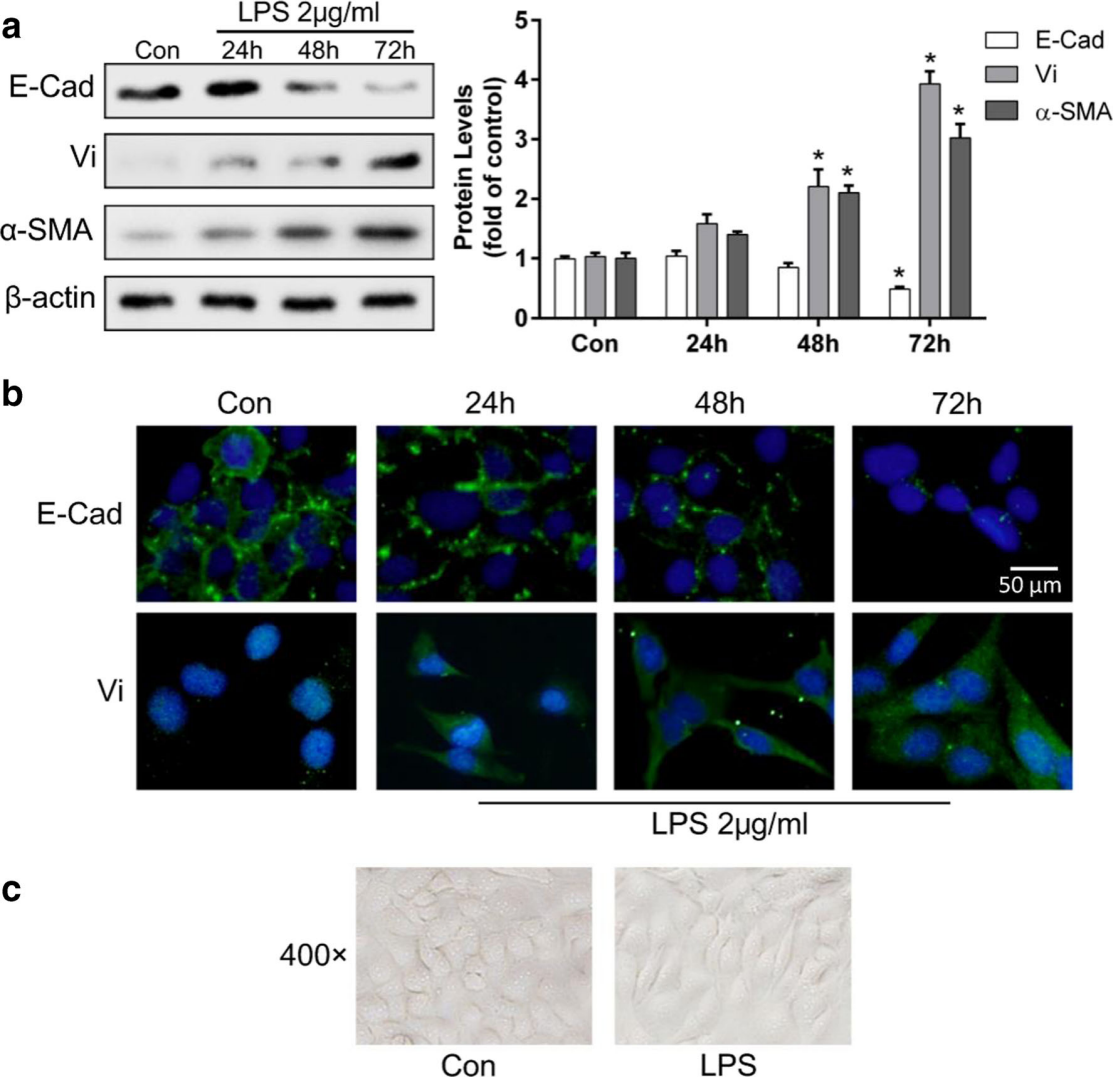
*P. aeruginosa*’s LPS-induced EMT in BEAS-2B cells. BEAS-2B cells were incubated with 2 μg/mL *P. aeruginosa*’s LPS for 24, 48, or 72 h. Western blotting revealed that LPS decreased the expression of E-Cad and upregulated the expression of Vi and α-SMA in a time-dependent manner (**a**). Immunofluorescence showed similar results of the upregulated mesenchymal marker Vi and downregulation of the epithelial marker, E-Cad, in a time-dependent manner (**b**). Compared with the basal condition, a fibroblast-like, spindle-shaped morphology was induced by *P. aeruginosa*’s LPS for 72 h in BEAS-2B cells (**c**). The data represent the mean ± SEM, *n* = 3. *p < 0.05 versus the control

Previous studies demonstrated that BEAS-2B cells could undergo EMT while exposed to many stimuli (Doerner and Zuraw 2009; Gulino et al. 2016; Polimeni et al. 2016); here we showed that *P. aeruginosa*’s LPS could induce EMT in BEAS-2B cells. After incubation with *P. aeruginosa*’s LPS (2 μg/mL), the expression of the epithelial marker, E-Cad, and mesenchymal markers, Vi and α-SMA, in BEAS-2B cells were detected using Western blotting at different time points (Fig. 1a). Meanwhile, immunofluorescence was performed to examine the expression of E-Cad and Vi after LPS incubation (Fig. 1b). It was showed that *P. aeruginosa*’s LPS significantly decreased the expression of the epithelial marker E-Cad and increased the expression of the mesenchymal markers, Vi and α-SMA, in a time-dependent manner by Western blotting. The immunofluorescence assay showed similar changes of E-Cad and Vi expressions in BEAS-2B cells treated with *P. aeruginosa*’s LPS. Furthermore, we examined morphologic changes in BEAS-2B cells with inverted microscopy (Fig. 1c). The results showed that compared with the control group, a fibroblast-like, spindle-shaped morphology was adopted in BEAS-2B cells incubated with 2 μg/mL *P. aeruginosa*’s LPS for 72 h. These data indicate that *P. aeruginosa*’s LPS was sufficient to induce EMT in BEAS-2B cells.

### *P. aeruginosa*’s LPS increased the expression of integrin αvβ6 in BEAS-2B cells

**Fig. 2:**
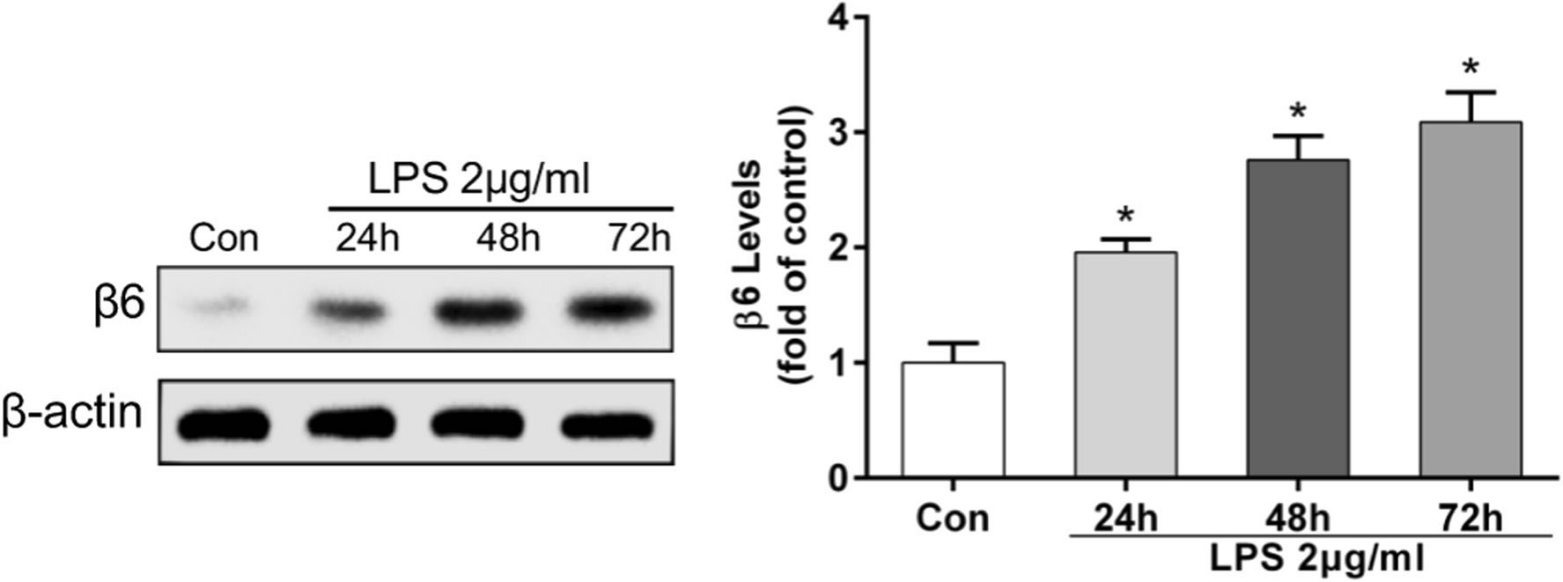
The expression of integrin αvβ6 in BEAS-2B cells was significantly upregulated by *P. aeruginosa*’s LPS in a time-dependent manner. BEAS-2B cells were exposed to 2 μg/mL *P. aeruginosa*’s LPS for 24, 48, or 72 h. Western blotting revealed that LPS increased the expression of integrin β6 in a time-dependent manner. The data represent the mean ± SEM, *n* = 3. *p < 0.05 versus the control

After incubation with *P. aeruginosa*’s LPS for 24, 48, 72 h, respectively, integrin β6 expression was examined using Western blotting. The results showed that the expression of β6 integrin was increased in a time-dependent manner (Fig. 2). Because integrin αvβ6 is the only heterodimer which comprise α and β subunits, and the upregulation of β6 is sufficient to increase surface expression of integrin αvβ6 (Niu et al. 2002), it was clear that *P. aeruginosa*’s LPS could induce an increase of αvβ6 expression in BEAS-2B cells significantly.

### Blocking integrin αvβ6 could reverse *P. aeruginosa*’s LPS-induced EMT in BEAS-2B cells

**Fig. 3:**
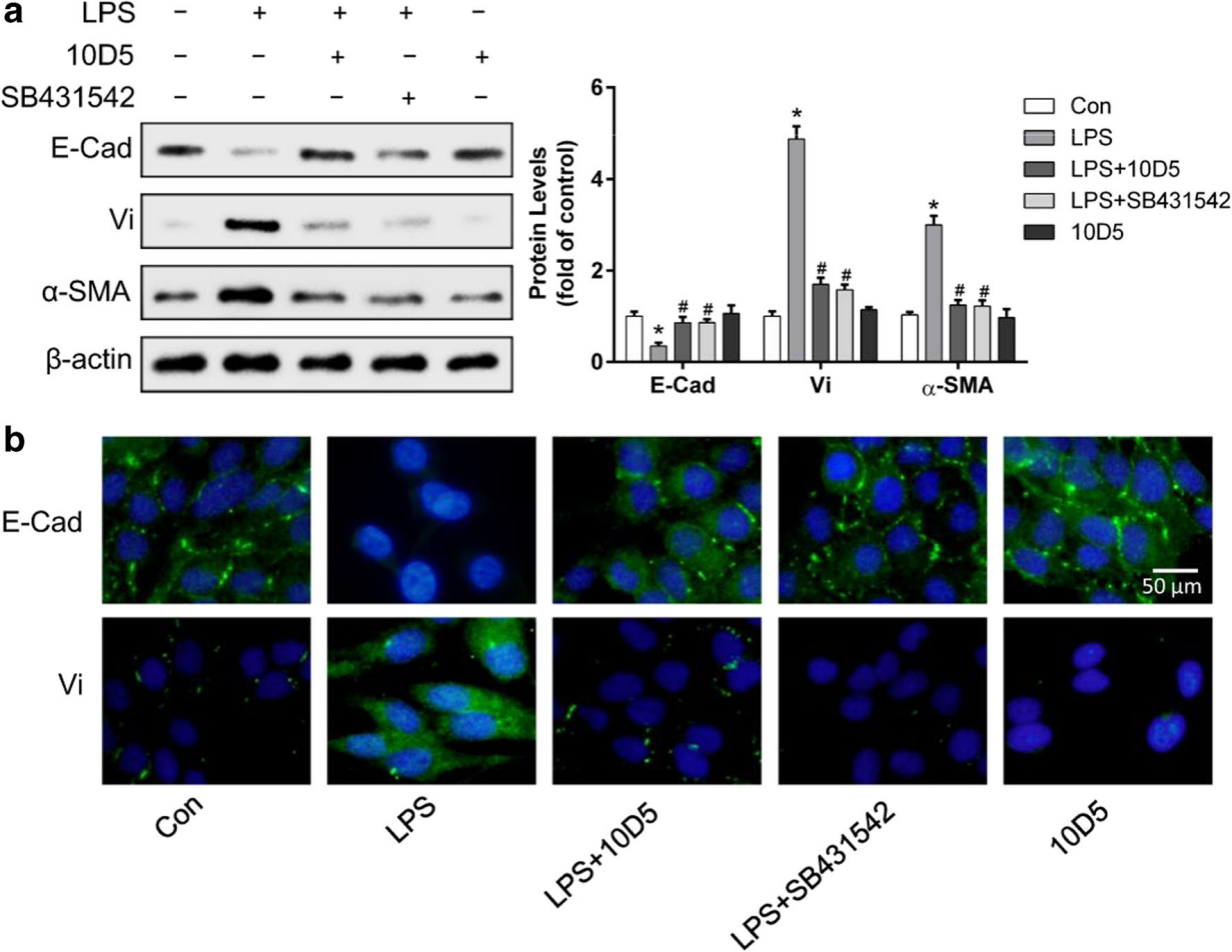
Blocking integrin αvβ6 could abrogate EMT in BEAS-2B cells induced by *P. aeruginosa*’s LPS. BEAS-2B cells were incubated with integrin αvβ6–blocking antibody 10D5 (30 μg/mL) or specific inhibitor of TGF-β1-Smad2/3 signaling, SB431542 (10 μM), for 2 h prior to incubation with 2 μg/mL *P. aeruginosa*’s LPS for 72 h. Western blotting showed that the decrease of the epithelial marker, E-Cad expression, and the increase of mesenchymal markers, Vi and α-SMA, induced by *P. aeruginosa*’s LPS were reversed by integrin αvβ6–blocking antibody 10D5 as well as SB431542 (**a**). Immunofluorescence showed similar results of E-Cad and Vi expressions (**b**). The data represent the mean ± SEM, n = 3. *p < 0.05 versus the control (Con), #*p* < 0.05 versus LPS

BEAS-2B cells were pretreated with integrin αvβ6–blocking antibody 10D5 or specific inhibitor of TGF-β1-Smad2/3 signaling, SB431542, at 10 μM for 2 h prior to incubation with LPS for 72 h. The expression of E-Cad, Vi, and α-SMA was detected using Western blotting (Fig. 3a). The expression of E-Cad and Vi was examined by immunofluorescence staining (Fig. 3b). The results showed that the LPS-induced decrease of E-Cad expression was significantly attenuated by integrin αvβ6–blocking antibody 10D5, while the increased expression of Vi and α-SMA was alleviated. These data indicated that blocking of integrin αvβ6 could inhibit the EMT process induced by *P. aeruginosa*’s LPS in BEAS-2B cells, which provided evidence that integrin αvβ6 plays an important role during *P. aeruginosa*’s LPS-induced EMT process in human airway epithelial cells. Furthermore, the findings demonstrated that TGF-β1-Smad2/3 signaling pathway may be critical for the pathologic process in BEAS-2B cells which could be inhibited by the specific TGF-β1 inhibitor SB431542.

### Integrin αvβ6 mediated *P. aeruginosa*’s LPS-induced EMT in BEAS-2B cells via TGF-β1-Smad2/3 signaling pathway

**Fig. 4:**
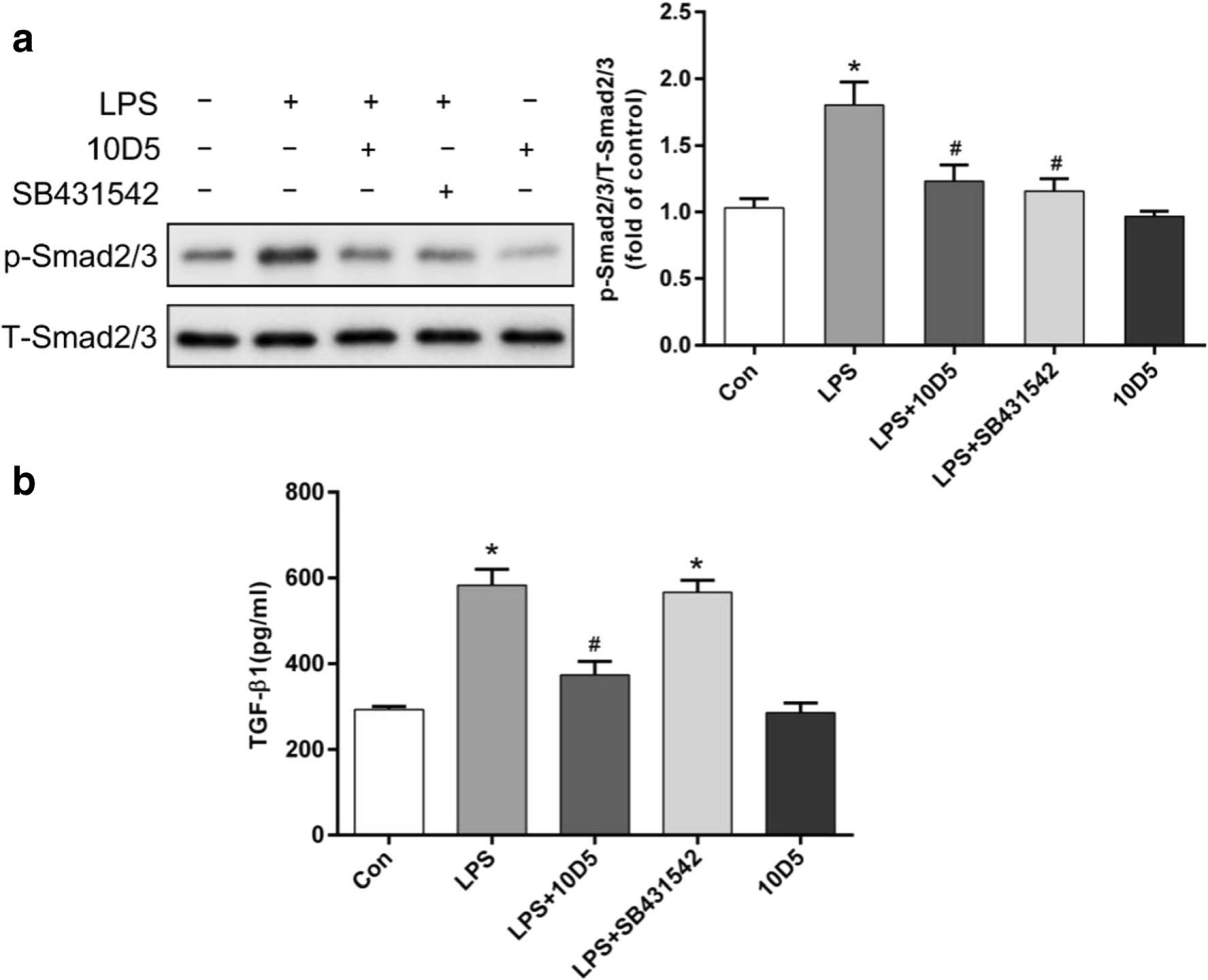
Integrin αvβ6 mediated *P. aeruginosa*’s LPS-induced EMT in BEAS-2B cells via TGF-β1-Smad2/3 signaling pathway. BEAS-2B cells were pretreated with integrin αvβ6–blocking antibody 10D5 (30 μg/mL) or TGF-β1 inhibitor, SB431542 (10 μM), for 2 h prior to incubation with 2 μg/mL *P. aeruginosa*’s LPS for 72 h. Total-Smad2/3 and p-Smad2/3 were detected using Western blotting (**a**). The secretion of active TGF-β1 in the culture medium was measured by ELISA (**b**). The results showed that the phosphorylation of Smad2/3 was increased by *P. aeruginosa*’s LPS, which were ameliorated by integrin αvβ6–blocking antibody 10D5 as well as SB431542. In addition, elevated secretion of active TGF-β1 was induced by *P. aeruginosa*’s LPS, which could be inhibited by integrin αvβ6–blocking antibody 10D5. The data represent the mean ± SEM, *n* = 3. *p < 0.05 versus Con, #*p* < 0.05 versus LPS

It has been shown that epithelium-specific αvβ6 could locally regulate the activity of TGF-β1, which mediates EMT and fibrosis via Smad2/3 signaling (Kim et al. 2006; Flier et al. 2010; Marmai et al. 2011). Thus, we examined phosphorylated Smad2/3 using Western blotting and measured the secretion of TGF-β1 using ELISA. *P. aeruginosa*’s LPS significantly increased the level of phosphorylated Smad2/3. Remarkably, integrin αvβ6–blocking antibody 10D5 could inhibit the increased level of p-Smad2/3 as well as the inhibitor of TGF-β1-Smad2/3 signaling pathway (Fig. 4a). The ELISA showed that increased secretion of active TGF-β1 in *P. aeruginosa*’s LPS stimulated BEAS-2B cells, which was decreased by αvβ6-blocking antibody 10D5 but not SB431542 (Fig. 4b). These results indicated that *P. aeruginosa*’s LPS-induced EMT in BEAS-2B cells was mediated by integrin αvβ6 via activation of TGF-β1-Smad2/3 signaling pathway.

### Blocking integrin αvβ6 could inhibit morphologic changes and the increase of MMP-2 and -9 secretion induced by *P. aeruginosa*’s LPS

**Fig. 5:**
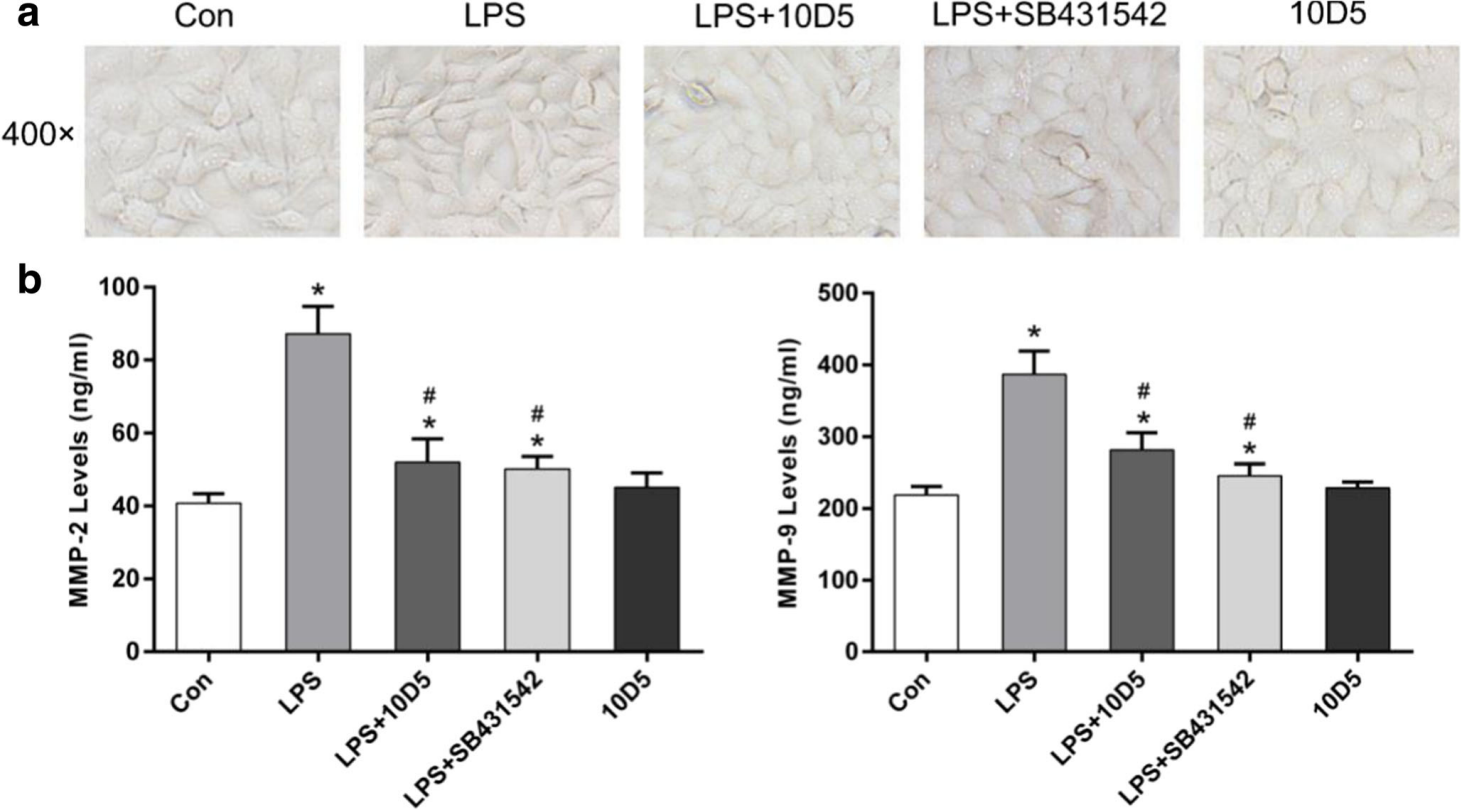
Blocking integrin αvβ6 could prevent morphologic changes and the increase of MMP-2 and -9 secretion induced by *P. aeruginosa*’s LPS. Cell morphologic change was ameliorated by 10D5 and SB431542 (**a**). ELISA showed that the secretion of active MMP-2 and -9 was significantly increased by *P. aeruginosa*’s LPS which could be attenuated by integrin αvβ6–blocking antibody 10D5 as well as specific inhibitor of TGF-β1-Smad2/3 signaling, inhibitor SB431542 (**b**). The data represent the mean ± SEM, *n* = 3. *p < 0.05 versus Con, #*p* < 0.05 versus LPS

Incubated with *P. aeruginosa*’s LPS at 2 μg/mL for 72 h, BEAS-2B cells adopted a fibroblast-like, spindle-shaped morphology instead of the original cobblestone-shaped appearance. Pretreatment with integrin αvβ6–blocking antibody 10D5 or TGF-β1 inhibitor, SB431542, could prevent the morphologic changes induced by *P. aeruginosa*’s LPS (Fig. 5a). Increased secretion of active MMP-2 and -9 induced by *P. aeruginosa*’s LPS treatment was detected using ELISA, which could be attenuated by both 10D5 and SB431542 (Fig. 5b).

## Discussion

*P. aeruginosa* is one of the major pathogens in patients with chronic airway diseases, such as cystic fibrosis, chronic obstructive pulmonary disease, bronchiectasis, and bronchiolitis obliterans (Moore and Mastoridis 2017). As a repairing response, airway fibrotic remodeling has been frequently observed in these patients, which could result in irreversible decline of pulmonary function and increased mortality. The mechanism of *P. aeruginosa*–associated airway remodeling remains unclear. In this study, we demonstrated that EMT could play a crucial role in *P. aeruginosa*–associated airway remodeling, which is regulated by epithelium-specific integrin αvβ6.

LPS, one of key virulence traits of *P. aeruginosa* to mediate the interaction between the bacterium and its host (Pier 2007; Raoust et al. 2009), was used to establish an EMT model in BEAS-2B cells. We evaluated EMT in BEAS-2B cells induced by *P. aeruginosa*’s LPS with multiple approaches to elucidate cell morphologic changes, the expression of epithelial and mesenchymal proteins, and the secretion of MMPs. The results showed that *P. aeruginosa*’s LPS significantly increased the expression of mesenchymal markers, Vi and α-SMA, accompanied with a decrease of the epithelial marker E-Cad. Meanwhile, BEAS-2B cells stimulated by *P. aeruginosa*’s LPS changed their cobblestone-like morphology into a fibroblast-like, spindle-shaped morphology. Additionally, increased secretion of MMP-2 and -9 was induced by *P. aeruginosa*’s LPS in cell medium. Thus, we demonstrated that *P. aeruginosa*’s LPS is sufficient to induce EMT in human airway epithelial cells. And the findings suggest that EMT is an important source of fibroblasts and plays similar roles during *P. aeruginosa*–associated airway fibrotic remodeling as it does in pulmonary and renal fibrosis (Hahm et al. 2007).

TGF-β1 has been considered a key inducer of EMT and has a central role in regulating fibrosis process (Kalluri and Neilson 2003; O’Connor and Gomez 2014). Previous studies revealed that *P. aeruginosa* could significantly increase the secretion of TGF-β1 in vivo (Botha et al. 2008; Vos et al. 2008; Cigana et al. 2016). Our data revealed that *P. aeruginosa*’s LPS could increase the secretion of TGF-β1 in cell medium of BEAS-2B, which suggested that TGF-β1 may have a critical role in *P. aeruginosa*–associated airway fibrosis remodeling. Additionally, the level of phosphorylated Smad2/3 was significantly elevated during *P. aeruginosa*’s LPS-induced EMT in BEAS-2B cells, while SB431542, a selective inhibitor of TGF-β1-Smad2/3 signaling pathway, reversed the changes of EMT markers’ expression and secretion of MMPs as well as morphologic alteration. Previous studies showed that TGF-β1 could induce EMT by mediating Smad-dependent and Smad-independent signaling pathways (Doerner and Zuraw 2009; Gulino et al. 2016; Polimeni et al. 2016); our data indicated that Smad-dependent signaling pathway was the predominant mechanism involved in *P. aeruginosa*’s LPS-induced EMT in BEAS-2B cells, although Smad-independent signaling pathway may also contribute to the pathologic process.

Even with the critical role of TGF-β1 in the fibrosis process, blocking TGF-β1 globally could increase the risks of severe systemic side effects, such as systemic inflammation, immune disorders, tumors, and even death (Flavell et al. 2010; Seoane and Gomis 2017), which could lead to complicated conditions in infectious patients. It may be an ideal choice to inhibit TGF-β1-Smad2/3 signaling locally for intervention in airway fibrotic remodeling.

Integrin αvβ6 can bind to latency-associated protein of the inactive TGF-β1 complex and provide spatially restricted activation of TGF-β1 (Munger et al. 1999; Horan et al. 2008; Puthawala et al. 2008; Katsumoto et al. 2011). It has been reported that blocking αvβ6 could prevent fibrosis in multiple organs including the lungs without systemic side effects (Wang et al. 2007). Whether integrin αvβ6 is involved in *P. aeruginosa*–associated airway fibrotic remodeling remains unclear. Our data here showed that *P. aeruginosa*’s LPS significantly increased integrin αvβ6 expression in BEAS-2B cells, which was consistent with previous finding that the expression of integrin αvβ6 could be dramatically upregulated in response to epithelial cell injury or inflammation (Breuss et al. 1995). Furthermore, we used blocking antibody 10D5 to identify the regulatory effect of integrin αvβ6 on EMT in BEAS-2B cells induced by *P. aeruginosa*’s LPS. Western blotting revealed that *P. aeruginosa*’s LPS decreased the expression of the epithelial marker E-Cad and increased the expression of mesenchymal markers, Vi and α-SMA, which could be alleviated by 10D5. The immunofluorescence assay showed similar results of EMT markers’ expression. These data indicated that blocking integrin αvβ6 could attenuate *P. aeruginosa*’s LPS-induced EMT in BEAS-2B cells, which supports our hypothesis.

Although the studies from Kim and Wang revealed that integrin αvβ6 could regulate EMT in vivo and in vitro via activation of TGF-β1-Smad2/3 signaling pathway (Kim et al. 2006; Flier et al. 2010; Marmai et al. 2011), whether *P. aeruginosa*–associated airway fibrotic remodeling depends on the interaction between integrin αvβ6 and TGF-β1-Smad2/3 signaling is currently unknown. In this study, BEAS-2B cells were pretreated with 10D5 at 30 μg/mL for 2 h and then incubated with *P. aeruginosa*’s LPS for 72 h. We detected the secretion of active TGF-β1 using ELISA and measured the phosphorylation level of Smad2/3 using Western blotting, respectively. According to our results, *P. aeruginosa*’s LPS increased TGF-β1 secretion in cell medium and upregulated the phosphorylated Smad2/3 in BEAS-2B cells, which could be ameliorated by blocking integrin αvβ6. As similar effects of SB431542 were detected on the in vitro model, these results indicated that integrin αvβ6 could regulate *P. aeruginosa*’s LPS-induced EMT in BEAS-2B cells via activation of TGF-β1-Smad2/3 signaling pathway.

It is noteworthy that toll-like receptor 4 (TLR4), a receptor for LPS which could enhance TGF-β signaling pathway, has been reported to play a key role in the process of organ fibrogenesis and LPS-induced EMT process(Guillot et al. 2004; Seki et al. 2007; He et al. 2016; Tang et al. 2018; Vidya et al. 2018). And interestingly, previous studies implicated that cross talks exist between toll-like receptors and integrin αvβ6(Pittet et al. 2013; Jolly et al. 2014), so integrin αvβ6 might be a therapeutic target for toll-like receptor-mediated fibrogenesis process. Our findings that blocking integrin αvβ6 could ameliorate *P. aeruginosa*’s LPS-induced EMT in BEAS-2B cells provided another strong evidence for the above hypothesis.

In this study, we also examined whether integrin αvβ6 could regulate morphologic changes and MMP secretion induced by *P. aeruginosa*’s LPS. Our results showed that *P. aeruginosa*’s LPS induced BEAS-2B cells to transdifferentiate into a fibroblast-like, spindle-shaped phenotype instead of the original cobblestone-like morphology, which could be alleviated by integrin αvβ6–blocking antibody 10D5. The finding is consistent with a previous study that integrin αvβ6 can regulate morphologic changes during EMT process (Ramos et al. 2009).

MMP-2 and -9 have been demonstrated to be important contributors to fibrosis process by activating TGF-β1 and degrading basement membranes (Corbel et al. 2001; Corbel et al. 2002); here we showed that elevated secretion of MMP-2 and -9 was induced by *P. aeruginosa*’s LPS, which were significantly attenuated by blocking integrin αvβ6. These data indicated that integrin αvβ6 could regulate MMP-2 and -9 secretion accompanied with EMT in BEAS-2B cells, which was consistent with the studies from Ahmed et al. (2002) and Thomas et al. (2001).

## Conclusion

**Fig. 6:**
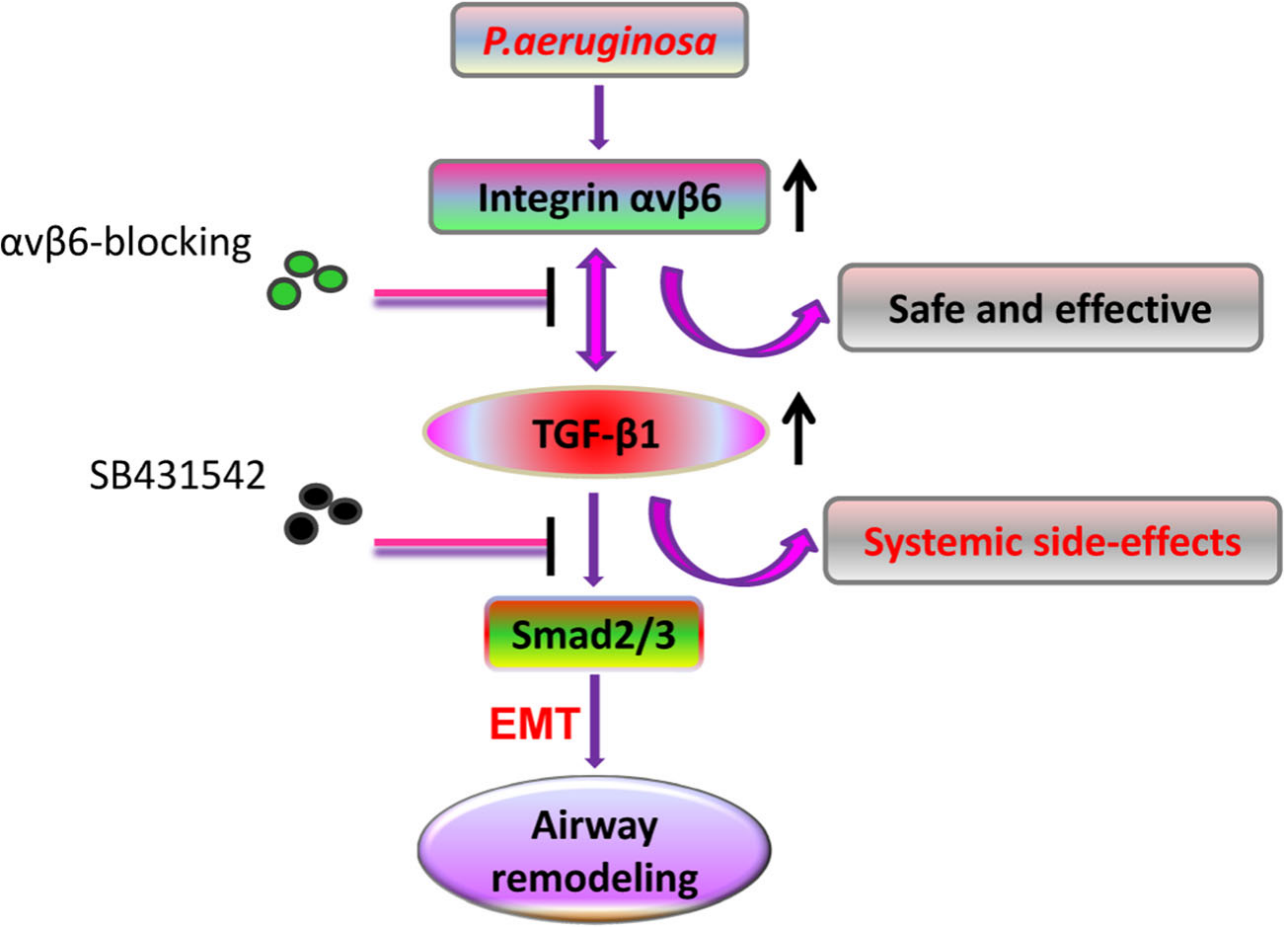
Summary. Integrin αvβ6 could regulate EMT in BEAS-2B cells induced by *P. aeruginosa*’s LPS via activation of TGF-β1-Smad2/3 signaling pathway. Compared with globally blocking TGF-β1, integrin αvβ6 blocking might be a more promising option to prevent airway remodeling induced by *P. aeruginosa*

In summary, this study demonstrated that *P. aeruginosa*’s LPS is sufficient to induce EMT in human bronchial epithelial cells which in turn could contribute to airway fibrotic remodeling. Furthermore, our results obtained here suggest that *P. aeruginosa*’s LPS-induced EMT in BEAS-2B cells could be regulated by integrin αvβ6–mediated TGF-β1-Smad2/3 signaling activation, and blocking αvβ6 could abrogate EMT changes of BEAS-2B cells, including expression of EMT markers, cell morphology, and secretion of MMP-2 and -9. Thus, integrin αvβ6 might be a safe and effective therapeutic target for *P. aeruginosa*–associated airway remodeling (Fig. 6).
